# Omics Sciences in Regular Physical Activity

**DOI:** 10.3390/ijms26125529

**Published:** 2025-06-10

**Authors:** Rosamaria Militello, Simone Luti, Alessandra Modesti

**Affiliations:** Department of Experimental and Clinical Biomedical Sciences, University of Florence, 50134 Florence, Italy; rosamaria.militello@unifi.it (R.M.); simone.luti@unifi.it (S.L.)

**Keywords:** multiomics, exercise, systems biology, exercise adaptation

## Abstract

The multiple health benefits of regular physical activity are well known and are the results of exercise adaptations. The study of physical training biology is not straightforward since it involves organ crosstalk and depends on numerous variables, such as type of exercise or individual physiology. A multiomic approach allows us to analyze proteins, metabolites, lipids, and epigenetic modifications on a wide scale, so it is a valid tool to identify numerous patterns and clarify how exercise exerts its beneficial effects. Stimuli given by physical activity lead the body to re-establish a new dynamic balance at the level of redox homeostasis and metabolic state. Evaluating the effect of specific training is important for maximizing the beneficial effects of physical activity. In this review we provide a brief overview of different omics technologies used in this field. For each “omics” we analyzed studies published in the last 10 years and highlighted the main molecules identified with that approach. We then described future challenges in their application from the perspective of using new bioinformatics and artificial intelligence tools.

## 1. Introduction

According to the World Health Organization (WHO), physical activity (PA) is any bodily movement produced by skeletal muscles that requires energy expenditure.

**Figure 1:**
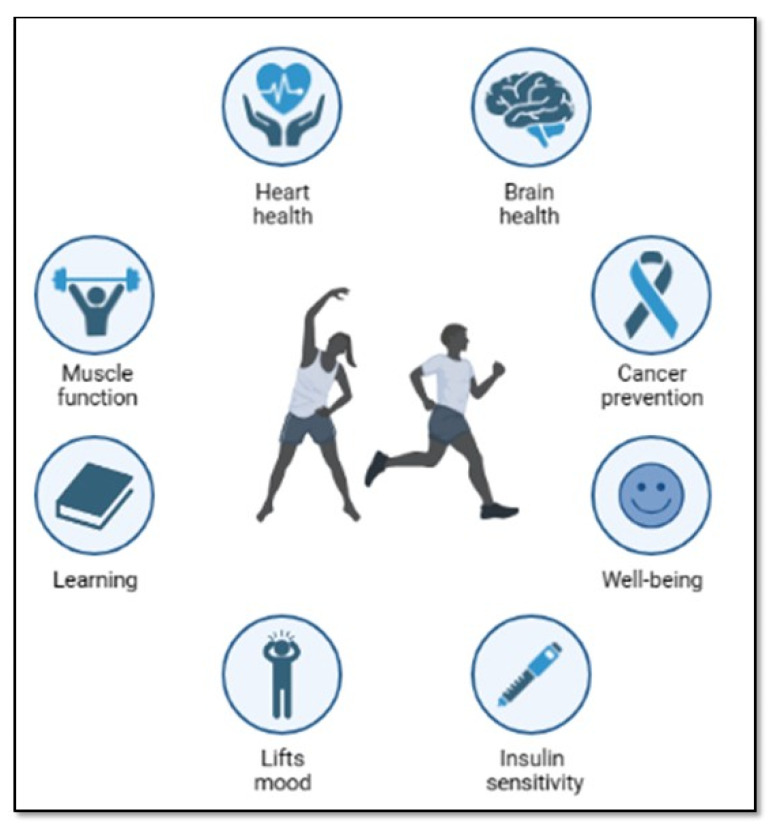
Health benefits of regular physical activity.

Exercise training is defined as planned bouts of physical activity that repeatedly occur over a period lasting from weeks to years [1]. The multiple health benefits of regular physical activity are well known; some are reported in Figure 1 and have a long history. Hippocrates, the father of scientific medicine, was the first physician to recognize the value of exercise [2]. PA helps to prevent cardiovascular disease, diabetes, stroke, and breast and colon cancer. It also prevents overweight and obesity and improves mental health, quality of life, and well-being [3]. According to the WHO guidelines on physical activity for health, all adults should undertake 150–300 min of moderate-intensity, or 75–150 min of vigorous-intensity physical activity, or some equivalent combination of moderate-intensity and vigorous-intensity aerobic physical activity, per week [4].

However, exercise can have both positive and negative effects on inflammatory and redox status.

When the workload becomes excessive, it is easy to reach a state characterized by fatigue, lack of energy, and a sense of exhaustion. This acute phase is defined as “overreaching” and is reversible with an adequate rest period. In contrast, when it persists over time, it can lead to chronic fatigue syndrome (CFS) and overtraining syndrome, characterized by fatigue, an increase in pro-inflammatory markers, and a decrease in performance, with harmful consequences for health [5].

Early identification of fatigue through a combination of objective and subjective workloads measures allows coaches to prevent injury and provides a comprehensive picture of how athletes respond to training dose [6].

Physical training biology is complex to study because it involves numerous interactions among cells, tissues, organs, and systems, with remarkable crosstalk among them [7].

A multiomic approach allows for the analysis of proteins, metabolites, lipids, epigenetic modifications, and mRNA expression in a wide scale to identify numerous patterns and clarify how exercise exerts its beneficial effects.

The term “omics” refers to disciplines that use high-throughput technologies to produce large amounts of data. Omics sciences aim to identify, characterize, and quantify all biological molecules that are involved in a biological system of interest to obtain a snapshot of the underlying biology. Since 2001, when the human genome was sequenced, there has been an omics revolution thanks to the development of new technological approaches and bioinformatics tools [8]. Omics have been applied in various fields of biology, including the study of regular physical exercise and training. Currently, there is still much to learn about how physical activity can promote benefits for the body. The identification of specific markers, such as oxidative stress or signal molecules of inflammation, has become important for monitoring the level of training. This allows for the prevention of overtraining and helps adjust training loads to improve performance.

In this review, we summarized factors influenced by regular physical exercise and training. Moreover, we provided a brief overview of different omics technologies used in this field; for each “omics” we provided a systematic overview of studies published in the last 10 years and described future challenges in their application.

## 2. Bibliographic Search Method

**Figure 2:**
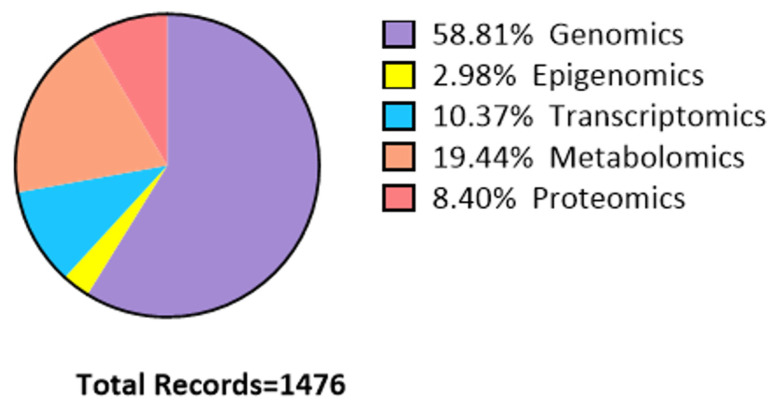
Pie chart showing the distribution of selected articles among various omics fields.

The bibliographic search was conducted using the PubMed database, with the keywords shown in Table 1. Papers published between 2014 and 2024 were selected. The search was restricted to free full text availability and English language. The filters “species: human” and “age: adult (19–44 years)” were then applied. The paper selection process is reported in Table 1, while the pie chart in Figure 2 represents the distribution of the selected papers across the various omics categories. Most of the selected papers (58.81%) are on genomics, while 2.98% address epigenomics. Furthermore, for each omics, a table is provided, which summarizes the content of the articles reported in the paragraph. These articles were selected according to the criteria shown in Table 1. To further reduce the complexity related to disease or nutritional status, papers were screened per title. Papers with titles containing the words “disease”, “illness”, “inflammation”, “syndrome”, “cancer”, “nutrition”, ”diet”, or “supplement” were excluded.

## 3. Role of Intensity, Volume, and Type of Activity on Regular Physical Exercise

Different types of physical activity are associated with different health outcomes by activating metabolic and signaling pathways.

The following paragraph highlights how different types of physical activity, as well as intensity and duration, can influence health outcomes, thereby emphasizing the complexity of the topic.

Evidence suggests that the benefits obtained from exercise are related to its intensity and duration. Warburton et al. report that, in most studies, a linear dose–response relationship between PA and health benefits is not observed; in fact, the rate of incremental gains in health benefits may be attenuated at higher PA volumes [9].

Acute and chronic training are two different exercise paradigms. Acute exercise is defined as a single session, while chronic exercise or exercise training refers to repeated sessions over either a short-term or long-term period. Acute or short-term exercise activates both insulin-dependent and insulin-independent mechanisms, whereas longer-term effects involve “organ crosstalk”, such as communication from skeletal muscle to adipose tissue, liver, and pancreas, which mediates beneficial systemic effects on blood pressure, blood glucose levels, and serum lipid profiles [10].

Furthermore, muscle contraction and physiological functions during training require energy, which depends on exercise intensity and duration. An acute bout of exercise leads, in the short term, to the activation of glycolysis [11], while in the long-term adaptations involve lipolysis and the mobilization of hepatic glycogen stores [12], making glucose and free fatty acids available as source of energy.

**Figure 3:**
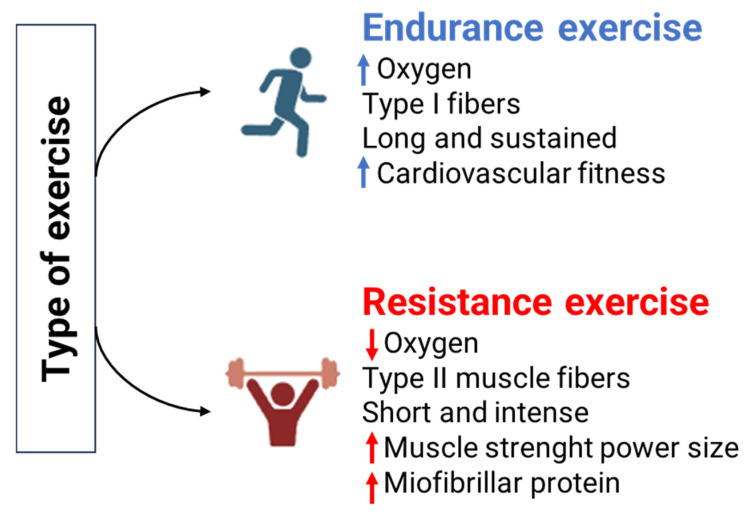
Main differences between endurance and resistance exercise. An upward arrow represents an increase, whereas a downward arrow indicates a decrease.

Two fundamental types of physical activity can be distinguished: endurance exercise (EE) and resistance exercise (RE), although a continuum exists between these modalities (Figure 3).

Endurance exercise, such as walking, cycling, and jogging is performed to improve cardiovascular fitness, while resistance or strength training improves muscle strength, power, size, and endurance [13].

At muscle level, EE can increase oxidative capacity secondary to increased mitochondrial density and thus mitochondrial protein. Acute endurance exercise induces multiomic changes related to energy metabolism, oxidative stress and immune response. Later adaptations involve pathways related to energy homeostasis, tissue repair, and/or remodeling [14]. Resistance exercise training results in increases in strength and muscle fiber cross-sectional area (CSA), particularly in myofibrillar proteins myosin and actin [15]. Table 2 reports the studies analyzed in this review regarding aerobic and anaerobic exercise, along with their main findings and key molecules involved.

## 4. Role of Exerkines on Regular Physical Exercise

As seen in the previous paragraph, different types of training protocols produce different outcomes by activating metabolic and signaling pathways. This paragraph highlights how physical exercise induces changes in body through the release of molecules, emphasizing the role of exerkines.

**Figure 4:**
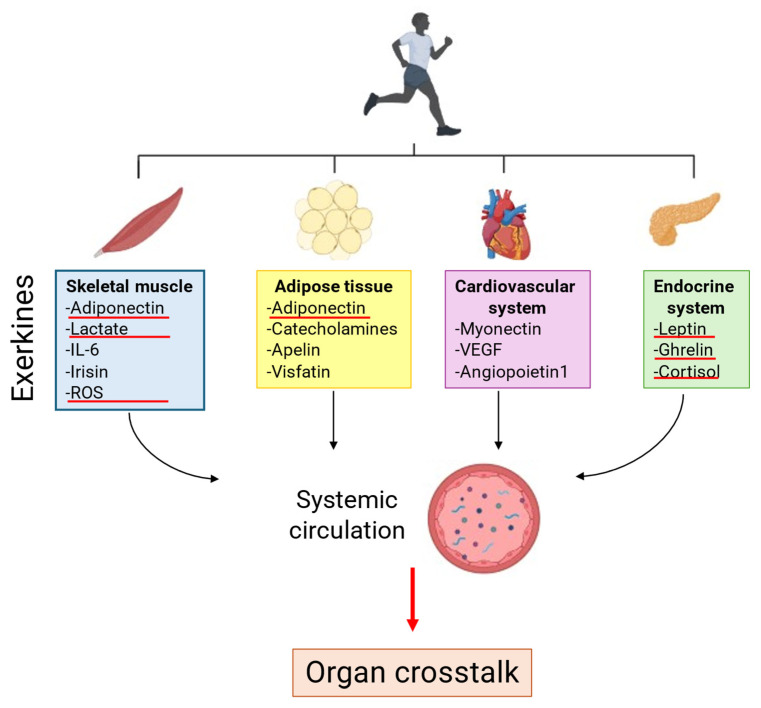
Exerkines released through crosstalk between different tissues and organs.

Regular physical exercise induces the release of molecules into circulation that are collectively called “exerkines”. The term “exerkines” refers to the totality of all humoral exercise factors (peptides and RNA species) that are expressed, produced, and secreted by various tissues and organs. These molecules act to promote crosstalk between organs and enhance the systemic benefits of exercise [10]. The main important exerkines are shown in Figure 4.

Tissue crosstalk occurs through extracellular vesicles (EVs) [16]. It is known that EVs transport and transfer their cargo (protein, lipid, and microRNAs) to other cells [17] and are therefore closely associated with exerkines. The metabolic demands during physical activity vary between tissues and the trafficking of vesicles enables the sharing of resources, thereby reducing energy consumption, protein translation, and degradation [18]. The release of EVs is generally associated with increases in intracellular calcium (Ca^2+^). Motor neuron stimulation is one phenomenon that involves the rapid release of Ca^2+^ from the sarcoplasmic reticulum, so the release of EVs may be associated with it [18].

Among exerkines are myokines that are released by muscle cells. Skeletal muscle, in fact, acts as an endocrine organ, releasing signaling molecules such as cytokines and peptides, which play a crucial role in communication between muscles and other tissues. To date, the biological functions of only 5% of all known myokines have been described [19]. Myokines mediate communication between muscle and other organs, including the brain, adipose tissue, bone, liver, and muscle itself. This supports the concept that inter-tissue signaling is essential for training. Myokines are involved in muscle proliferation, differentiation, and regeneration, as well as mediating energy supply [20,21].

An important myokine is lactate, which is present in the blood at millimolar concentrations [22]. Its physiological concentration varies among individuals, typically ranging from 1.5 to 7.5 mM and can rise to 10–40 mM in inflammatory environments [23]. Lactate, derived from muscle contraction [24], was one regarded as a metabolic waste product and a cause of muscle fatigue. However, it is now recognized as a mediator of training and a signal molecule, leading to the proposed term “lactormone” due to its considerable blood distribution and systemic effects [25,26]. Lactate operates both at rest and during exercise and plays important roles in metabolism, redox biology, mitochondrial biogenesis, and epigenetics.

Interleukin 6 (IL6) is the most extensively studied myokine. It acts as an energy sensor in skeletal muscle during exercise, promoting increased hepatic glucose output and enhanced glucose uptake in muscle cells [27].

Evaluating the effects of specific training programs on myokine release and metabolic status is valuable, as it could be used to monitor both long- and short-term effects during training exercise.

In this context, the use of omics sciences represents a powerful approach for the monitoring and discovery of myokines and exerkines in general. Table 3 represents the studies analyzed in this review regarding exerkines and their main findings and the key molecules involved.

## 5. Omics Sciences

Omics sciences aim to identify, characterize, and quantify all biological molecules involved in a biological system of interest. Here, we provide a brief overview of how different omics approaches have been used to study the effects of regular physical exercise, focusing on genomics, epigenomics, transcriptomics, metabolomics, and proteomics.

### 5.1. Genomics

Recent advances in technologies such as DNA sequencing, gene-editing, and big data processing have enabled the analysis of a large number of genomes [28]. Various projects, such as ELITE, GAMES, Gene SMART, GENESIS, and POWERGENE have been developed to discover genomics- and post-genomics-based biomarkers [29] with the aim of identifying talent, predicting the risk of sports-related injuries and maximizing physical performance outcomes [28]. As of the end of May 2023, 251 DNA polymorphisms have been associated with athlete status, of which 128 genetic markers were positively associated with athlete status in at least two studies [30]. The field of sports genomics has identified several single nucleotide polymorphisms (SNPs) that are associated with athletic status and a wide range of physical activity behaviors [31].

Several studies have shown that angiotensin-converting enzyme (ACE) insertion/deletion (I/D) gene polymorphisms play a significant role in determining athletic abilities. The ACE I allele may favor endurance performance, while the ACE D allele may predispose individuals to power/strength performance. Additionally, the actinin Alpha 3 (ACTN3) gene has been studied for its association with athletic performance. Studies suggest that the ACTN3 R allele may provide an advantage in power/sprint events [32].

Association studies have also identified a genetic predisposition to musculoskeletal soft tissue injuries. These injuries have been linked to genes encoding components of the extracellular matrix in musculoskeletal soft tissue. Genetic variations in the collagen COL5A1 and COL1A1 genes have been associated with soft tissue injuries, endurance running performance, range of motion, and exercise-associated muscle cramping [33]. Table 4 presents the studies analyzed in this review regarding genomics, along with their main findings and the key genes or molecules involved.

### 5.2. Epigenomics

**Figure 5:**
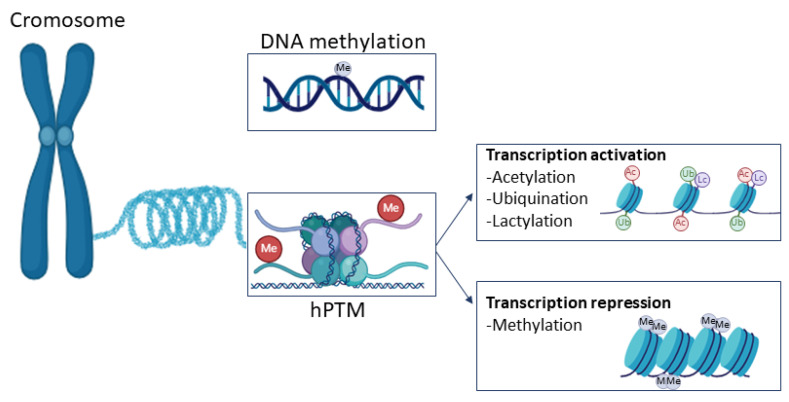
Epigenetic mechanisms: DNA methylation and histone post-translational modifications (hPTM):methylation (Me, blue dots); Acetylation (Ac, red dots); Ubiquitination (Ub, green dots); Lactylation (Lc, purple dots).

Epigenetics can be defined as the study of mitotically and/or meiotically heritable changes in gene function that cannot be explained by changes in DNA sequence. Epigenome is highly dynamic and undergoes continuous regulation through epigenetic modifications. These modifications include DNA methylation and histone post-translational modifications (hPTMs) (Figure 5), and it has been demonstrated that regular physical activity can modulate gene expression through these mechanisms [34]. Table 5 presents the studies analyzed in this review regarding epigenomics, along with their main findings and the molecules involved.

#### 5.2.1. DNA Methylation

DNA methylation is the most extensively studied epigenetic mechanism in skeletal muscle in relation to exercise [35]. It involves in the addition of a methyl group to 5′-positions of cytosine residues at cytosine-guanine dinucleotide (CpG) islands by DNA methyltransferases. Hypermethylation makes DNA less accessible to DNA-binding proteins and RNA-polymerases, leading to transcriptional repression [36]. Baseline differences in skeletal muscle DNA methylation related to sex have been reported [37]. Changes in DNA methylation can be induced by both acute exercise and chronic training [36]. A single bout of exercise leads the reduction in DNA methylation and the activation of the genes involved in mitochondrial function and fuel utilization, including peroxisome proliferator-activated receptor gamma coactivator 1-alpha (PGC1α) and pyruvate dehydrogenase kinase isozyme 4 (PDK4) [38]. Endurance-trained athletes, compared to resistance-trained and untrained individuals demonstrate different DNA methylation patterns in selected skeletal muscle genes after a bout of acute exercise. While some of these differences relate to muscle fiber-type distribution, slow-twitch fiber-type genes such as MYH7 and MYL3 show lower promoter methylation and higher expression in endurance-trained athletes. Conversely, this group exhibited higher methylation in transcription factors such as FOXO3, CREB5, and PGC-1α [39]. In general, physical activity appears to prevent hypermethylation in skeletal muscle [40].

#### 5.2.2. Histone Post-Translational Modification

Histone post-translational modifications (hPTMs) such as acetylation, methylation, phosphorylation, and ubiquitination are epigenetic mechanism that regulate histone binding to DNA. These modifications alter the chromatin structure and influence various chromatin-dependent processes [34].

Metabolites generated by metabolic pathways act as cofactors or substrates for PTMs, which, in histones, occur primarily on lysine, arginine, and serine residues [34,41]. The relationship between epigenetic modifications and metabolism is bidirectional, with acetylation being the best-characterized example [41].

A single session of acute exercise has been shown to cause a global increase in histone H3 acetylation [42]. Additionally, a single bout of endurance exercise leads to the phosphorylation of class II histone deacetylases (HDACs), resulting in reduced nuclear abundance and increased MEF2 (myocytes enhancer factor 2) DNA-binding activity in skeletal muscle [42]. MEF2 is a transcription factor whose activity is normally repressed by class II histone deacetylases (HDACs).

Recently, it has been discovered that lactate is also involved in post-translational modification. Zhang et al. (2019) identified the lactylation of lysine 18 on histone (H3K18) [43]. Similar to other post-translational modifications, lactylation affects transcriptional regulation and, similarly to acetylation, activates gene expression [44]. The acetyltransferase p300 has been shown to increase both H3K27 acetylation and H3K18 lactylation, although the process by which it is regulated is still not clear [45].

To date, its role in exercise physiology is still not completely clarified.

**Table 5:** Selected articles concerning epigenomics.

Author	Year	Aerobic or Anaerobic Exercise	Main Findings	Main Molecules Identified
Dimauro et al. [34]	2020	Both	Exercise influences DNA methylation and histone modifications.	Histones, DNA methylation, and redox-related molecules during exercise.
Jacques et al. [35]	2019	Both	DNA methylation and histone changes in human skeletal muscle following exercise.	DNA hypomethylation marks genes such as PGC-1a, TFAM, MEF2A, and PDK4, along with histone modifications such as H3K36 and H3K9/14 acetylation.
Voisin et al. [36]	2015	Both	Exercise training affects DNA methylation.	p15, ASC, PGC-1α, PDK4, TFAM, PPAR-δ, citrate synthase, MEF2A, MYOD1.
Landen et al. [37]	2023	Both	Physiological and molecular sex differences in muscle response to exercise training.	DNA methylation of hormone-related transcription factors as the androgen receptor and the estrogen receptor.
Fabre et al. [38]	2018	Both	DNA methylation in human adipose tissue after exercise.	DNA methylation in ADRA2A, FOSL1, METRNL, RARA, CBLB, GPR132, and RELT.
Geiger et al. [39]	2024	Both	DNA methylation of exercise-responsive genes differs between trained and untrained individuals.	Exercise-responsive genes MYH7 and MYL3, high methylation in transcription factors such as FOXO3, CREB5, and PGC-1α.
Turner et al. [40]	2020	Both	DNA methylation of HOX genes.	Hypermethylated state in genes KIF15, DYRK2, FHL2, MRPS33, ABCA17P, and HOX genes.
Li et al. [44]	2020	Both	Glis1 induces epigenome-metabolome signaling cascade influenced by exercise.	Glis1 and modulation of glycolytic gene expression.

### 5.3. Transcriptomics

Transcriptomics represents the link between the genome and the proteome. The transcriptome is composed of coding messenger RNAs (mRNAs), ribosomal RNA (rRNA), transfer RNA (tRNA), and a variety of noncoding RNAs such as small RNAs (sRNAs) [46]. The principal techniques used for transcriptome analysis are essentially three: real-time RT-PCR, microarrays, and next-generation RNA sequencing (RNA-seq) [47]. Unlike the genome, the transcriptome is highly dynamic and changes in response to different factors. In the past, the study of transcriptomics was based exclusively on the study of mRNAs, but the presence of non-protein coding genes in the human genome has changed the status of non-coding RNAs making them important for the regulatory network of gene expression [48]. Furthermore, miRNAs are characterized by their stability in the blood and by their key role in organ cross-talk. These characteristics make them a valid subject for research in the exercise field. Several studies, in fact, underline their ability to reflect performance and the type of exercise [49] (Table 6).

Long non-coding RNAs (lncRNAs) do not code for proteins but regulate gene expression, finely tuning nuclear architecture and transcription in the nucleus, as well as controlling the stability of mRNA [50]. They represent a novel biomarker for regular physical exercise [51]. Physical activity also regulates genes through miRNAs, small single-stranded non-coding RNA molecules involved in RNA silencing and post-transcriptional modulation of gene expression. For example, miR-140-3p is differentially expressed in athletes’ blood after exercise [52]. Other miRNAs have been found to correlate with some physiological parameters such as VO2 max [53] or are crucial in skeletal muscle development.

ROS generated during exercise can regulate epigenetic machinery by modulating DNA, histone modifications, and ncRNA transcripts. Epigenetic modulators affect the expression of genes involved in generating, sensing and neutralizing enzymes that impact the cellular levels of ROS and RNS. However, the causal link between epigenetic modifications and exercise-induced ROS is not completely established [34].

**Table 6:** Selected articles concerning transcriptomics.

Author	Year	Aerobic or Anaerobic Exercise	Main Findings	Main Molecules Identified
De Sanctis et al. [51]	2021	Aerobic	Non-coding RNAs differentiating in skeletal muscles in elderly trained in endurance vs. resistance exercise.	Non-coding RNAs: miR-20a-5p and miR-106-5p shared 108 mRNA involved in the regulation of TGF-beta, p53, FoxO, Hippo, WNT, MAPK, and HIF-1.
Hecksteden et al. [52]	2016	Aerobic	Non-coding RNAs in sports training.	Non-coding RNAs hsa-miR 513b-5p, hsa-miR-140-5p, hsa-miR-650, and hsa-miR 3620-3p regulate vascular endothelial growth factor A pathway.
Domańska-Senderowska et al. [53]	2019	Both	MicroRNA profiles and their adaptive response to exercise training.	Non-coding RNAs microRNA-1, -21, -23a, -124, -125b, -133a/b, -144, -145, -206, -486, and -696 regulate the IGF1/PI3K/AKT/mTOR signaling pathway.
Dimauro et al. [34]	2020	Both	Exercise in redox homeostasis and epigenetic regulation in skeletal muscle.	Non-coding RNAs: miR-1, miR-16, miR-21, miR-26a, miR-29a, miR-126, miR-133a, miR-133b, and miR-206, miR-210, miR-221, 328, miR-378, miR-451, miR-494, miR-21, miR-221, miR-20a, miR-146a, miR-133, and miR-222.

### 5.4. Metabolomics

Metabolomics is the omics science that studies small molecule chemical entities with molecular weights less than or equal to 1500 Daltons [54]. It provides a picture of the metabolic state of the biological system under study and its use has increased in different research areas, including exercise physiology.

Exercise plays a crucial role in regulating cellular metabolic pathways, making metabolomics a valuable tool in sports research. This has led to the introduction of the concept of “sportomics” [55]. In the last decade, metabolomics has been applied to different sports, including soccer, basketball, and rugby. Metabolomics allows a qualitative and quantitative analysis of different metabolites in biological samples using various platforms based on mass spectrometry, such as capillary electrophoresis–mass spectrometry (CE-MS), liquid chromatography–mass spectrometry (LC-MS), ultra-performance liquid chromatography–mass spectrometry (UPLC-MS), gas chromatography–mass spectrometry (GC-MS), or nuclear magnetic resonance (NMR) spectroscopy [56].

Two types of metabolomics investigations can be distinguished: targeted and untargeted. Targeted analysis identifies and quantifies metabolites of interest in specific biological processes/pathways. The untargeted analysis quantifies all detectable metabolites in a semi-quantified way to detect new biomarkers [55].

One branch of metabolomics is lipidomics and includes eight lipid categories: glycerolipids, glycerophospholipids, sphingolipids, sterol lipids, prenol lipids, saccharolipids, and polyketides [57]. Oxylipins are the products of the oxidation of polyunsaturated fatty acid (PUFA) from n6 and n3 [58]. These molecules have different regulatory roles, such as immune function and inflammation [59]. Exercise could influence their generation. For example, both acute and chronic exercise can release oxylipids that are stable enough to be measured in plasma or muscles several hours after training [60,61]. The advances in mass spectrometry allow the identification of many oxylipids whose role in exercise will be the subject of future research [57].

Metabolomics allows the analysis of several metabolites in different biological samples [62], and the use of each of them presents both advantages and disadvantages, as reported in Table 7.

Several studies have focused on blood to measure metabolites in order to investigate adaptations and responses to acute and chronic exercise [63]. However, saliva is an alternative for studying the molecules involved in exercise adaptation [64]. The measurement of total salivary content may provide a non-invasive, low-stress method for assessing exercise-associated metabolic, hormonal, and immunologic status and for evaluating exercise load. Recently, many studies have used saliva to assess concentrations of these compounds in response to exercise and training [65], and its composition reflects systemic health status [64]. The major limitation of salivary samples is the inter-individual variability, which may affect salivary composition, thus making comparison among individuals difficult [66]. Despite these limitations, it is interesting to monitor the concentration changes of salivary metabolites that occur during strenuous exercise. Pitti et al. reported an altered concentration of 56 metabolites after a football match in the saliva of female athletes [67]. The first application of metabolomics to soccer was published by Santone et al. in 2014 [63]. Since then, sportomics has continued to grow, generating a large amount of data. To analyze all these data, the use of bioinformatic tools is required, such as Kyoto Encyclopedia of Genes and Genomes (KEGG) pathways, Reactome, MetaboAnalyst. Undoubtedly, a standardized protocol for data collection and analysis would help to improve the comparability among different studies [68].

In the future, sportomics could be used to prevent muscular damage and fatigue in athletes. Moreover, metabolomics applied to sports could identify several biomarkers of oxidative stress, muscular damage, and energy deficit [55]. Sportomics could be a complementary tool to the current methods of studying and monitoring athletes’ state. However, the high cost of the purchase, maintenance, and the demand for trained personnel to operate the machinery are still the major problems for these types of analyses in exercise and sport science. Table 8 reports the studies analyzed in this review regarding metabolomics and their main findings and molecules involved.

### 5.5. Proteomics

Since protein composition represents the functional status of a biological system, exercise proteomics allows the identification of pathways that change with training. This makes it possible to also identify and monitor (new) proteins that offer insights into athletes’ condition to prevent the overtraining/overreaching syndrome and improve sports performance. Proteomics complexity is caused by protein post-translational modifications. According to classical proteomic techniques, proteins must be first fractionated by a combination of differential detergent, separated by two-dimensional gel electrophoresis (2DE) and digested into peptides before MS identification [69]. Mass spectrometry-based proteomics has become an emerging tool for systems biology and has also been applied to exercise and sports sciences [70]. The main limitations of proteomics are the cost of equipment and the need for software tools to analyze large data sets. Despite that, it is a highly complementary tool to genomic approaches such as microarray analysis and allows the evaluation of training-induced modifications from a qualitative and quantitative perspective. Franco-Martinez et al. found sex-specific differences between men and women in the salivary proteome at rest and after acute exercise and concluded that sex is the factor that most strongly modulates salivary protein content [71]. According to Balfoussia et al., athletes taking part in Spartathlon express plasma proteins that are differentially expressed and involved in inflammation, antioxidant, coagulation, and the transport of Vitamin D and iron pathways [72]. Exercise is also able to model PTM as acetylation [73] and sumoylation [74]. Magherini et al., for example, found that the levels of protein carbonylation were reduced after acute swimming bursts in rats [75]. Proteomics contributes to uncovering the molecular mechanisms of exercise adaptation in muscle cells. Strength training activates the PI3K-Akt-mTOR pathway, resulting in protein synthesis and muscle growth. In contrast, aerobic training inhibits protein synthesis through the regulation of AMPK-TSC2 which downregulates mTOR [76]. Phosphorylation-mediated signaling pathways are fundamental for cells to respond to stimuli. The MoTrPAC project allows the cataloging of phosphoproteomic alterations in response to different modes of exercise. These results could shed light on the exercise-induced signaling events [77]. Table 9 reports the studies analyzed in this review regarding proteomics, along with their main findings and the molecules involved.

## 6. Bioinformatic Tools

The large amount of data that emerge from omics techniques require powerful software tools for management and analysis [78].

The term multiomics refers to analyses that integrate diverse omics data. The interpretation of a single omics dataset cannot explain the complex interactions that occur in biological systems, which consequently need integrated multiomics analysis to be explored in depth [79]. The most common and simple method of integrating two different omics datasets is the correlation or co-mapping, for example, between the transcriptome and the proteome [80]. This method could be used even by researchers with relatively weak computing skills, while multiomics requires complex algorithms and functional interpretation beyond the validation of findings [81]. Fortunately, open-source tools and shared databases are available to help non-computational researchers. The European Molecular Biology Laboratory (EMBL), a leading international organization in the life sciences, includes research units, services and facilities operating in six sites across Europe. In particular, the EMBL’s European Bioinformatic Institute (https://www.ebi.ac.uk/research; accessed on 4 June 2025) deals with the management of big biological data.

They provide freely available data and bioinformatic services such as a unified query system that quickly finds omics data according to keywords [82] or different kinds of training.

Other tools worth mentioning are reported in Table 10: MetaboAnalyst 6.0 (https://www.metaboanalyst.ca, accessed on 4 June 2025), MergeOmics 3.0 (https://mergeomics.research.idre.ucla.edu, accessed on 4 June 2025), Cytoscape (https://cytoscape.org, accessed on 4 June 2025), InCroMAP (www.cogsys.cs.uni-tuebingen.de/software/InCroMAP, accessed on 4 June 2025), and 3Omics (https://3omics.cmdm.tw, accessed on 4 June 2025). A detailed description of other tools is reported in the reviews of Chen et al., 2023 and Cambiaghi et al., 2016 [78,81]. The huge amount of data, combined with the variety of research topics, brings with it the problem of annotation and storage; high performance computing and cloud-based solutions are needed to achieve fast multiomics data processing [83].

Although omic sciences have been widely applied to the study of human exercise, there is a lack of integration of various omics in human samples. Based on our knowledge, a notable example of successful multiomic integration in exercise science is the Molecular Transducers of Physical Activity Consortium (MoTrPAC). This initiative created the first multiomic, whole-organism map of the temporal effects of eight weeks of endurance exercise training in male and female Rattus norvegicus and profiled the temporal transcriptome, proteome, metabolome, lipidome, phosphoproteome, acetylproteome, ubiquitylproteome, epigenome, and immunome in whole blood, plasma, and 18 solid tissues [84].

Briefly, the main computational barriers of omics in healthcare are data volume and complexity, data quality, and lack of standardization, all of which make it difficult to create a multiomic human map. The management of big data, in fact, requires statistical rigor and tools to interpret and represent the results in a reliable and friendly way, due to the complexity and heterogeneity caused by the different technologies used for their acquisition [85]. Bioinformatics training that includes both statistical analysis and programming is fundamental for handling such data in a correct way [86].

The integration with the emerging artificial intelligence techniques will play a crucial role in integrative analysis, interpretation, and visualization [87]. Benefits obtained from its use concern not only the identification of hidden patterns but also the prediction of biological outcomes [88], leading to the development of personalized training protocols. However, numerous challenges remain, despite the progress of AI, such as data quality and the need for experts to implement AI approaches [87].

## 7. Conclusions

To conclude, the development of multiomic technologies in the last ten years has allowed the study of the effects of regular physical activity and the identification of the pathways and molecules involved in this process.

Sports coaches and exercise physiologists could use this knowledge to assist athletes more efficiently using targeted exercises, as well as to maximize health benefits induced by training.

However, differences in the applied physical activity protocols (intensity, duration, and type of exercise), the choice of sampling time points, which could mask the effect of exercise on circulating levels of biomarkers [89], the subject’s physiology and age, as well as the small sample size, increase the difficulty of studying the body’s adaptation to exercise. To resolve these limitations, the Molecular Transducers of Physical Activity Consortium (MoTrPAC), whose aim is to generate a molecular map of exercise adaptations, uses both animal and human [90] studies. Despite that, the translation of knowledge obtained with a multiomics approach into the application of personalized training plans remains a challenge for the future. The use of Artificial Intelligence will be fundamental in the coming decades to address this challenge and to overcome practical barriers to the adoption of omics in sports and public health. In fact, the lack of trained personnel to operate the necessary machinery used in omics and analyze the obtained data remains one of the major obstacles to the spread of this techniques. However, this problem can be overcome by the use of artificial intelligence. Actually, the guidelines for therapeutic exercise are focused on outcomes for healthy adults without taking into account what intensity and volumes are more beneficial for certain populations [91]. The development of omics technologies and bioinformatic tools, combined with greater collaboration among health professionals will emphasize personalized training. The future role of omics is, therefore, to support the development of individualized training programs.

## 8. Limitations

One of the main limitations of this work is the exclusion of articles whose titles contained terms such as “disease,” “illness,” “inflammation,” “syndrome,” “cancer,” “nutrition,” “diet,” or “supplement.” Although this choice significantly limited the state of the art considered in the review, it was necessary due to the large amount of literature, which would have otherwise made it difficult to produce an effective synthesis.

Furthermore, we decided to focus the analysis exclusively on humans aged between 19 and 44 years, thus concentrating on the adult population. This decision was driven by the aim of minimizing the influence of other factors, such as the hormonal instability typical of adolescence or the comorbidities more common in older age, which could have affected the results related to the effects of physical activity.

## Figures and Tables

**Table 1:** Selection process of relevant studies.

Keywords	Total	2014–2024	Free Full Text	English	Humans	19–44 Years Old	Records After Duplicate Remove
Omics AND exercise	392	349	282	281	145	30	40
Omics AND PA	509	456	362	360	187	40	
Genomics AND exercise	7135	4901	3527	3520	2163	634	868
Genomics AND PA	12,178	7510	5441	5427	3126	845	
Epigenomics AND exercise	281	251	178	177	133	34	44
Epigenomics AND PA	426	380	282	277	199	43	
Transcriptomics AND exercise	1760	1340	1057	1057	481	129	153
Transcriptomics AND PA	2391	1826	1427	1426	643	150	
Metabolomics AND exercise	1768	1605	1188	1183	653	221	287
Metabolomics AND PA	2328	2133	1592	1585	854	286	
Proteomics AND exercise	1328	1069	781	778	359	102	124
Proteomics AND PA	1981	1574	1160	1153	536	122	

**Table 2:** Selected articles concerning aerobic and anaerobic exercise.

Author	Year	Aerobic or Anaerobic Exercise	Main Findings	Main Molecules Identified
Warburg et al. [9]	2017	Both	Health benefits, cardiovascular improvements, and longevity.	Not specific to molecules, focused on general health benefits and physiological effects of exercise.
Nederveen et al. [10]	2020	Both	Role of extracellular vesicles and exosomes in exercise science in cell communication and adaptation.	Exosomes, extracellular vesicles, and various proteins involved in cellular communication post-exercise.
Nayor et al. [11]	2020	Both	Metabolic response to acute exercise in middle-aged adults.	Metabolites: amino acids, lipids, glucose, lactate, and several hormones involved in metabolic response.
Aldiss et al. [12]	2018	Aerobic	Influence of exercise on adipose tissue.	Brown adipose tissue markers, UCP1 (uncoupling protein 1), and other adipokines involved in thermogenesis.
Morville et al. [13]	2020	Both	Metabolic shifts based on exercise type.	Metabolites: amino acids (e.g., BCAA), fatty acids, glucose, and other metabolites linked to exercise metabolism.
Kim et al. [14]	2022	Both	Genetic factors influencing human performance to endurance and resistance exercise.	Genes: PPARs (peroxisome proliferator-activated receptors), FNDC5 (fibronectin type III domain-containing protein 5), and ACE (angiotensin-converting enzyme).
Roberts et al. [15]	2024	Anaerobic	Molecular signatures in human skeletal muscle in response to resistance training.	Proteins: MYH (myosin heavy chains), actin, PGC-1α (peroxisome proliferator-activated receptor gamma coactivator), IGF-1 (insulin-like growth factor 1).

**Table 3:** Selected articles concerning exerkines.

Author	Year	Aerobic or Anaerobic Exercise	Main Findings	Main Molecules Identified
Nederveen et al. [10]	2020	Both	Role of extracellular vesicles in tissue communication and adaptation.	Exosomes, extracellular vesicles, miRNAs, and proteins involved in cell signaling.
Sabaratnam et al. [16]	2022	Both	Exercise-induced organ crosstalk involving muscle.	Myokines (e.g., IL-6, irisin), adipokines, and cytokines involved in muscle–organ interactions.
Thomou et al. [17]	2017	Both	Circulating miRNAs from adipose tissue regulate gene expression in distant tissues.	miRNAs from adipose tissue, including miR-30a-5p, miR-27a-3p, and miR-155.
Whitham et al. [18]	2018	Both	Extracellular vesicles as mediators of tissue crosstalk during exercise.	Extracellular vesicles, miRNAs, proteins like IL-6, and adipokines.
Severinsen et al. [19]	2020	Both	The emerging roles of myokines in muscle–organ crosstalk, inflammation, metabolism, and muscle growth.	Myokines (e.g., IL-6, irisin, FGF21), and adipokines influencing metabolism.
Pourteymour et al. [20]	2017	Aerobic	Identification of novel exercise-regulated myokines of human skeletal muscle.	Myokines: IL-6, irisin, FGF21.
Catoire et al. [21]	2014	Both	Identification of exercise-induced myokines and their regulatory role in metabolism.	Myokines: IL-6, irisin, FGF21, and other factors regulating fat metabolism and muscle growth.
Chow et al. [22]	2022	Both	Role of exerkines on released signaling molecules during exercise.	IL-6, FGF21, irisin, BDNF influencing metabolism, stress responses, and disease.
Pucino et al. [23]	2017	Aerobic	Lactate as a key molecule released during exercise.	Lactate.
Sá Filho et al. [24]	2023	Both	Lactate as mediator on mental health.	Lactate, neurotransmitters, and miRNAs involved in brain-muscle communication.
Brooks et al. [25]	2022	Both	Lactate as a myokine influencing metabolism during exercise.	Lactate, IL-6, FGF21, and other myokines.
Pal et al. [27]	2014	Both	Role of IL-6 in metabolic regulation after exercise.	IL-6, signaling molecules related to muscle metabolism.

**Table 4:** Selected articles concerning genomics.

Author	Year	Aerobic or Anaerobic Exercise	Main Findings	Main Molecules Identified
Tanisawa et al. [28]	2020	Both	Genetic factors influencing physical performance and adaptation to exercise.	ACE, PPARA, ACTN3, VEGF.
Pitsiladis et al. [29]	2016	Both	Markers that predict athletic performance.	ACTN3, ACE, PPARA, VEGF, FTO.
Semenova et al. [30]	2023	Both	Genes involved in athletic performance, endurance, strength, and recovery.	Genetic variants in ACTN3, ACE, BDNF, PPARA, and myostatin (MSTN).
Ahmetov et al. [31]	2016	Both	Polymorphisms in genes related to endurance and strength.	ACE, ACTN3, VEGF, FTO, myostatin, PPARA.
Durmic et al. [32]	2017	Anaerobic	Polymorphisms in ACE and ACTN3 genes in response to acute exercise.	ACE (Angiotensin-converting enzyme), ACTN3 (Alpha-actinin-3).
Wang et al. [33]	2017	Both	COL1A1 gene polymorphisms and their association with tendon and ligament injuries in athletes.	COL1A1 gene polymorphisms.

**Table 7:** Advantages and disadvantages of using biological samples for metabolomic analysis.

Sample	Advantages	Disadvantages
Blood	Allows the study of all endogenous metabolites secreted by different tissues and could be used for all methods of analysis.	Very invasive, difficult to evaluate the origin of metabolites identified, can be easily degraded due to the presence of enzymes in the sample.
Saliva	Easy to pick up and handle. Reflects the state of the body.	Could be affected by the condition of mouth, such as the presence of bacteria, and may show lower concentrations of endogenous metabolites compared to blood.
Urine	Easy to pick up and handle. Contains stable metabolites and both endogenous and exogenous compounds.	Could be affected by diet and external factors such as bacteria. The presence of urea and salts can be a problem for MS.
Tissue	Allows the study local metabolites detectable at high concentrations	Very invasive, limitation in the amount of sample that can be taken.

**Table 8:** Selected articles concerning metabolomics.

Author	Year	Aerobic or Anaerobic Exercise	Main Findings	Main Molecules Identified
Bongiovanni et al. [55]	2019	Both	“Sportomics” linked to performance analysis.	Summary of the most important studies of metabolites related to exercise performance and biomarkers for sports performance.
Nieman et al. [57]	2020	Both	Exercise immunology.	Lipid and Krebs cycle metabolites, n-6/n-3PUFA oxylipins.
Vella et al. [60]	2019	Aerobic	Lipid profile responses to resistance exercise.	Lipid profiles: cyclooxygenase (COX)-derived thromboxanes and prostaglandins (PGE2), lipoxygenase (LOX), monohydroxy-eicosatetraenoic acids (5-HETE, 12-HETE, 15-HETE), monohydroxy-docosahexaenoic acids (4 HDoHE, 7-HDoHE, 14-HDoHE). CYP pathway-derived epoxy- and dihydroxy-eicosa trienoic acids (5,6-EpETrE, 11,12-DiHETrE and 14,15-DiHETrE).
Egan et al. [62]	2016	Aerobic	Exercise metabolism and its effects on muscle energy systems and performance.	Metabolites related to aerobic metabolism, lipid oxidation, glucose metabolism, and mitochondrial function.
Santone et al. [63]	2014	Both	Saliva metabolomics by NMR in sport performance.	Salivary metabolites, amino acids, lipids, and lactate, glucose, glycerol, and citrate.
Luti et al. [64]	2023	Both	Chronic training effects on metabolic and proteomic responses to exercise.	Salivary metabolites, amino acids (lysine, valine, glycine, tyrosine) citric acid, taurine.
Ntovas et al. [65]	2022	Both	Effects of physical exercise on saliva composition.	Salivary metabolites, biomarkers of exercise intensity (alpha amylase, salivary cortisol IgA, IgM, IgG), melatonin, lactate, testosterone, and oral health indicators.
Pitti et al. [67]	2019	Both	Salivary metabolome changes due to exercise-induced stress.	Exercise-induced metabolites: amino acids, acetate, creatine, dimethylamine, ethanol, ethanolamine, formate, fumarate, glycerol, lactate, ornithine.
Bongiovanni et al. [68]	2022	Both	Metabolomics in team-sport athletes.	Metabolites in urine and fecal samples: adenine, creatine, glutamine, carnitine, arachidonic acid, plasmalogen, cortisol, lysine, tyrosine, leucine, valine, and isoleucine, glutamate, beta-citryl-glutamate, 5-oxoproline, fatty acid-carnitines and acylated carnitines, trimethylamine-N-oxide (TMAO), dimethylglycine, O-acetyl carnitine, proline, betaine, acetoacetate, 3-hydroxy-isovaleric acid, acetone, N-methyl nicotinate, N-methyl nicotinamide, phenylacetylglutamine (PAG), 3-methylhistidine, trimethylamine (TMA), short-chain fatty acids, as well as methylamine, glycerate, allantoin, and succinate.

**Table 9:** Selected articles concerning proteomics.

Author	Year	Aerobic or Anaerobic Exercise	Main Findings	Main Molecules Identified
Franco-Martínez et al. [71]	2020	Anaerobic	Salivary proteome in response to acute exercise in men and women.	Men: keratin, ceruloplasmin, IgLambda, catalase, suprabasin, annexin A1.
Balfoussia et al. [72]	2014	Anaerobic	Plasma proteome under extreme physical stress.	Plasma proteins (albumin, fibrinogen), stress response proteins, oxidative stress biomarkers.
Gehlert et al. [74]	2016	Aerobic	Small ubiquitin-related modifier (SUMO)-1 in human myofibers.	SUMO-1 myofiber proteins: Lamina-A, actina, perinuclear region of myonuclei.
Gorini et al. [75]	2019	Both	Protein oxidation during exercise.	Oxidized plasma and muscle proteins (carbonylated proteins).
Gholamnezhad et al. [76]	2020	Both	Signaling pathways and muscle recovery.	Exercise adaptation-related proteins, signaling molecules (Rapamycin, myostatin/Smad) recovery biomarkers.
Wilson et al. [77]	2018	Both	Phosphoproteomics.	Phosphorylated proteins, signaling molecules (kinases).

**Table 10:** Main features of bioinformatic tools.

Tool	Main Functionality	Key Features	Best for
MetaboAnalyst	Web-based platform for comprehensive analysis of metabolomics data	- Data preprocessing - Statistical analysis - Pathway analysis - Visualization tools	Metabolomics data analysis and visualization
MergeOmics	Integration and analysis of multiomics data (e.g., genomics, proteomics, and metabolomics)	- Multiomics integration - Differential analysis - Pathway enrichment analysis	Multiomics data integration and analysis
Cytoscape	Open-source software for network analysis and visualization	- Network visualization - Integration of omics data into networks - Supports large networks	Network analysis, visualization, and biological data integration
InCroMAP	Network-based analysis for integrative omics data analysis (omics and clinical data)	- Multiomics integration - Clinical outcome prediction - Network analysis	Integrative omics and clinical data analysis
3Omics	Tool for the integrative analysis of multiomics data (omics, clinical, and phenotypic)	- Multiomics data integration - Predictive modeling - Pathway analysis - Data visualization	Integrative analysis of multiomics data with clinical information
